# Emtricitabine-induced pure red cell aplasia

**DOI:** 10.4102/sajhivmed.v20i1.983

**Published:** 2019-09-23

**Authors:** Nithendra Manickchund, Camille du Plessis, Melanie-Anne A. John, Thandekile C. Manzini, Bernadett I. Gosnell, Richard J. Lessells, Yunus S. Moosa

**Affiliations:** 1 King Edward VIII Hospital, Durban, South Africa; 2 Department of Infectious Diseases, University of KwaZulu-Natal, Durban, South Africa; 3 KwaZulu-Natal Research and Innovation Sequencing Platform (KRISP), University of KwaZulu-Natal, Durban, South Africa; 4 Centre for the AIDS Programme of Research in South Africa, University of KwaZulu-Natal, Durban, South Africa

**Keywords:** emtricitabine, pure red cell aplasia, drug induced, rare drug toxicity, adverse drug reaction, antiretroviral

## Abstract

**Introduction:**

Anemia is common in HIV. Parvo B19 infection is a well-recognised cause of red cell aplasia. Other causes of persistent pure red cell aplasia (PRCA) include anti-retroviral drugs such as zidovudine and lamivudine. We describe a case of PRCA that strongly implicates emtricitabine as the probable cause.

**Patient presentation:**

Patient was HIV positive and on treatment with a fixed drug combination consisting of tenofovir, emtricitabine and efavirenz for 3 months when she developed severe transfusion dependent anemia. The anemia, attributed to PRCA, was persistent and transfusion dependent for about one year.

**Management and outcome:**

Replacement of emtricitabine with abacavir resulted in a prompt, complete and lasting resolution of the anaemia, suggesting an etiologic role of emtricitabine in the PRCA.

**Conclusion:**

Emtricitibine is a rare cause of pure red cell aplasia.

## Introduction

There are 7.9 million people living with HIV in South Africa.^1^ Anaemia in the HIV infected is common, affecting 60% – 80% with advanced disease. There are several causes of anaemia, including HIV itself, opportunistic infections, malignancies, drug toxicities and malnutrition.^2^ Often a combination of factors contributes to anaemia.

Nucleoside reverse transcriptase analogues, such as zidovudine and lamivudine, are well-established causes of red cell aplasia.^3,4,5,6^ Considering the close structural and functional similarity between lamivudine and emtricitabine, it is not surprising that emtricitabine was recently implicated as a cause of pure red cell aplasia (PRCA) in a case series of four patients.^7^ We describe a case of PRCA that strongly implicates emtricitabine as the probable cause.

## Case report

A 35-year-old HIV-infected, pregnant woman developed a severe anaemia following initiation of anti-retroviral treatment (ART) consisting of a fixed drug combination of tenofovir, emtricitabine and efavirenz (Atroiza). She was diagnosed with HIV infection at 17 weeks gestation in August 2014 and was promptly started on ART. Her baseline CD4 cell count and haemoglobin (Hb) were 83 cells/mm^3^ and 8.2 g/dL respectively.

Three months later, she was admitted with a symptomatic, normochromic, normocytic anaemia with Hb of 2.2 g/dL and a normal white blood cell and platelet count. Haemolysis was excluded and an autoimmune screen was negative. There was no suggestion of ongoing blood loss. Electrolytes and liver function tests were normal. Epstein-Barr virus, Cytomegalovirus and hepatitis A, B and C serology were all negative. She received six units of packed red cells with post-transfusion Hb of 8.9 g/dL. She subsequently required an emergency caesarean section for foetal distress. Following surgery, her Hb repeatedly dropped, requiring frequent blood transfusions. A bone marrow aspirate and trephine biopsy, done 3 months later, revealed adequately represented myeloid series and megakaryocytes, but a marked reduction in erythropoiesis with maturation arrest at the pronormoblast stage. Overall findings were consistent with PRCA. Coupled with a positive qualitative serum parvovirus B19 (PVB19) polymerase chain reaction (PCR) (Parvovirus R-GENE® assay, Argene range, bioMérieux S.A., Verniolle France), a diagnosis of PVB19-induced PRCA was made even though giant pronormoblasts, which are pathognomonic for PVB19 marrow infection, were not observed.^8^ She was treated with a single course of 400 mg/kg/day of intravenous immunoglobulin (IVIG) over 5 days, but remained transfusion-dependent. The Department of Infectious Diseases was then consulted for further management. A second positive blood PVB19 qualitative PCR prompted continued treatment for PVB19-induced PRCA.

Over the following 11 months, she presented numerous times with symptomatic anaemia and received more than 53 units of packed red blood cells as well as six courses of IVIG at doses ranging from 0.4 mg/kg/day to 1 g/kg/day for 5 days. At no point did she demonstrate a reticulocyte response.^9^ A repeated bone marrow aspirate and trephine biopsy done 7 months after the first bone marrow examination did not contribute anything further. She remained adherent to ART and was virologically suppressed throughout.

**FIGURE 1:**
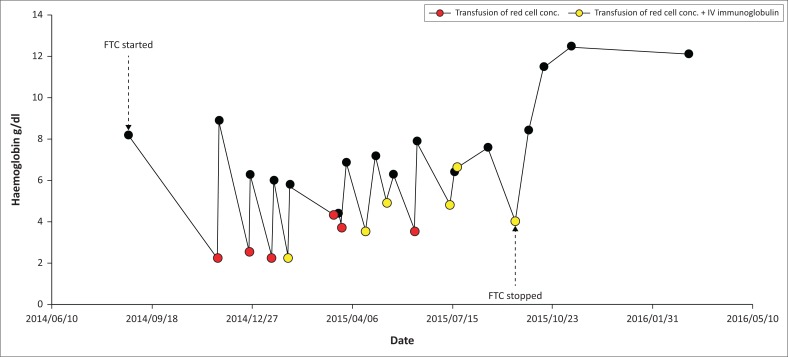
Changes in haemoglobin over time.

What was striking was that the problem of symptomatic transfusion requiring anaemia arose after starting ART, which suggested that the drugs could be playing a role. The close structural relationship between lamivudine and emtricitabine, and the rare but well-accepted fact that lamivudine is associated with PRCA, led us to implicate emtricitabine in this patient.^5,6^ A similar case of emtricitabine-induced PRCA was described in 2015 in a 39-year-old pregnant woman.^10^ We do not believe pregnancy had any role in the pathogenesis of PRCA in our patient because resolution occurred 1 year after delivery and promptly after discontinuing emtricitabine. Her ART regimen was changed to abacavir, tenofovir and efavirenz, resulting in a dramatic change in her condition. Her Hb spontaneously improved and she became transfusion-independent and remained so for the following 2 years of follow-up (Figure 1).

Interestingly, the qualitative PVB19 PCR remained positive a year after recovery of her Hb. We suspect our patient has asymptomatic PVB19 viremia, which has been described in < 1% of well blood donors.^11^

In summary, we describe a case of PRCA, initially presumed to be secondary to chronic PVB19 infection that failed to respond to multiple courses of IVIG, but promptly resolved after discontinuing emtricitabine. This case provides strong circumstantial evidence that emtricitabine played an etiologic role in the PRCA. This would be the fifth case of PRCA, implicating emtricitabine, reported from South Africa.^7^

### Ethical consideration

Ethics clearance was obtained from the Biomedical Research and Ethics Committee on 22 March 2016, since this report extended no risk to the patient. BREC reference number EXM188/16.
